# Measuring circulating miRNAs in early pregnancy could identify fetuses’ destined to undergrow and be at increased risk of stillbirth

**DOI:** 10.1016/j.ebiom.2020.103172

**Published:** 2020-12-16

**Authors:** Stephen Tong, Tu'uhevaha Kaitu'u-Lino

**Affiliations:** Translational Obstetrics Group, Department of Obstetrics and Gynaecology, University of Melbourne and Mercy Perinatal, Mercy Hospital for Women, Level 4, Mercy Hospital for Women, 163 Studley Rd., Heidelberg 3084, Victoria, Australia

## Commentary

As any reputable pregnancy app will attest, a human fetus grows terrifically while ensconced in mum's womb; swelling from the humble dimensions of a pomegranate seed at six weeks’ gestation to that of a sturdy 3.5 kilogram pumpkin some 7 months later. Healthy growth depends on a well-functioning placenta that keeps the fetus flushed with oxygen, well-nourished, and spirits away waste products.

In pregnancies complicated by placental insufficiency, fetal growth lags and the baby becomes small in utero; a condition called fetal growth restriction [1]. Being small in utero is a clinical indicator of placental insufficiency and is a stillbirth risk [1]. For instance, a fetus that is <10^th^ centile weight (adjusted for gestational age) incurs a 3-4 fold increased risk of demise while it remains in utero [2].

A first trimester screening test that points to pregnancies at increased risk of fetal growth restriction in later pregnancy would be clinically valuable. Such at risk pregnancies could be offered low dose aspirin which could reduce the risk of fetal growth restriction and possibly stillbirth [3]. Furthermore, increased surveillance by way of serial growth ultrasounds could be offered across pregnancy to promptly identify fetuses’ lagging in growth. This would allow timely diagnosis of fetal growth restriction and these pregnancies could be offered close monitoring with established clinical tests of fetal wellbeing, and timed birth (via an induction of labour or a caesarean section) before a stillbirth occurs [4].

Unfortunately, a first trimester screen test for fetal growth restriction with strong diagnostic test performance does not exist. A report by Kim et al [5] in this issue of *EBioMedicine* may have taken us closer to finding one. The team performed an unbiased screen of 800 miRNAs in plasma samples taken from the first half of pregnancy. These were obtained from 13 pregnancies where the final birthweight was <5^th^ centile (cases); and 16 pregnancies where the fetus was born with a normal birthweight (controls). To hunt for miRNA biomarkers, they used the nCounter miRNA profiling assay which deploys nanostring technology, which dispenses with an RNA amplification step and supposedly produces results with similar fidelity as qPCR. From this analysis they identified seven miRNAs that were differentially expressed in pregnancies where the fetus was destined to become growth restricted. Four of the seven miRs survived technical validation by qPCR.

The literature is replete with biomarker studies that lack validation in an independent cohort. Calculating diagnostic test performance simply from data derived from a large discovery screen results in ‘overfitting’ of the data, where the analysis reports over-optimistic diagnostic test performances (relative to how well it is likely to actually perform when applied to the general population). Hence, a strength of this study is that the team measured the same miRNAs in an independent, ‘validation’ cohort: 95 first trimester samples comprising 12 cases (<5^th^ centile birthweight) and 83 controls. Two circulating miRNAs remained significantly deranged among pregnancies destined to birth a small fetus: miR-374a-5p and miR-let-7d-5p.

Impressively, combining the two miRs produced a test with an AUC of 0.77. At a screen positive rate of 10% (where 10% who undertake the test will receive a high-risk result, a cut-off that is commonly used in pregnancy diagnostic studies), the sensitivity is around 60% (ie, the test identifies around 60% of growth restricted fetuses; see Figure 7 in Kim et al [5]). Remarkably, this pick-up rate is about on par with performing ultrasounds on every pregnancy during the last trimester of pregnancy (universal ultrasound) [6] and yet, this is potentially a test that can be applied around the very beginning of pregnancy. A sensitivity of 60% is far superior to the current clinical approach to identify small babies, which is to apply a tape measure on the abdomen to measure the uterine size and arrange an ultrasound for those measuring small (this picks up a paltry 20-30% cases) [6].

While the results in the validation study looks very good, it should be noted that a case control design can artificially inflate the apparent performance of a clinical test. The reason is that the incidence of ‘cases’ is far higher than what occurs in an unselected pregnant population (ie, the incidence of a baby <5^th^ centile should be around 5% rather than 12% (or 12/95)). Indeed, given the authors seemed to have the entire cohort in their biobank, they perhaps could have run the miRs in all their samples.

Nevertheless, this report is an enormously encouraging beginning. Hopefully the authors, or others, will further validate or extend these observations (such as finding other biomarkers that further lift the diagnostic performance of the two miRs).

Even if circulating miRs 374a-5p or let-7d-5p do not wind up being useful as a clinical biomarker (most biomarker candidates sadly disappoint when pressure tested in larger validation studies), it is conceptually interesting that these miRs are deranged as early as 12-14 weeks gestation in pregnancies destined to birth a small baby some 26-28 weeks later. It adds further evidence - first proposed just over 20 years ago [7] - that some cases of fetal growth restriction have their origins as early as the end of the first trimester, a sensitive moment in time when the small human is the mere size of a passionfruit.

## Declaration of Competing Interest

The authors are named inventors of a patent relating to the measurement of circulating SPINT1 as biomarker of placental insufficiency.
